# Modelling ligand exchange in metal complexes with machine learning potentials†

**DOI:** 10.1039/d4fd00140k

**Published:** 2024-08-03

**Authors:** Veronika Juraskova, Gers Tusha, Hanwen Zhang, Lars V. Schäfer, Fernanda Duarte

**Affiliations:** a Chemistry Research Laboratory, University of Oxford Oxford OX1 3TA UK fernanda.duartegonzalez@chem.ox.ac.uk; b Center for Theoretical Chemistry, Ruhr University Bochum D-44780 Bochum Germany lars.schaefer@ruhr-uni-bochum.de

## Abstract

Metal ions are irreplaceable in many areas of chemistry, including (bio)catalysis, self-assembly and charge transfer processes. Yet, modelling their structural and dynamic properties in diverse chemical environments remains challenging for both force fields and *ab initio* methods. Here, we introduce a strategy to train machine learning potentials (MLPs) using MACE, an equivariant message-passing neural network, for metal–ligand complexes in explicit solvents. We explore the structure and ligand exchange dynamics of Mg^2+^ in water and Pd^2+^ in acetonitrile as two illustrative model systems. The trained potentials accurately reproduce equilibrium structures of the complexes in solution, including different coordination numbers and geometries. Furthermore, the MLPs can model structural changes between metal ions and ligands in the first coordination shell, and reproduce the free energy barriers for the corresponding ligand exchange. The strategy presented here provides a computationally efficient approach to model metal ions in solution, paving the way for modelling larger and more diverse metal complexes relevant to biomolecules and supramolecular assemblies.

## Abstract 2

We introduce a strategy to train machine learning potentials using MACE, an equivariant message-passing neural network, for metal–ligand complexes in explicit solvents.

## Graphical abstract

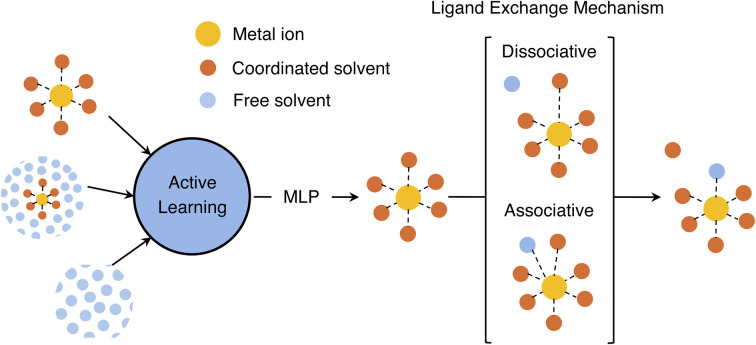

## 1 Introduction

Metal ions have a central structural and functional role in many molecular systems, including catalysts, supramolecular assemblies, and biomolecules. Due to their relevance, much work has been done to investigate the structure, kinetics, and thermodynamic stability of metal complexes in solution, including the dynamics of metal–ligand exchange reactions.^1^

Using a variety of experimental techniques, including X-ray absorption spectroscopy, neutron scattering and nuclear magnetic resonance (NMR) spectroscopy, several mechanisms have been proposed to describe ligand exchange in the first coordination shell of the metal ion. These mechanisms range from dissociative (D), involving an intermediate of lower coordination number, to associative (A), proceeding through an intermediate of higher coordination number. However, these are extreme cases – in most instances, no such idealised intermediate exists, and instead, a concerted interchange mechanism with dissociative (*I*_d_) or associate (*I*_a_) characteristics occurs.^2,3^

Of particular interest is ligand exchange with solvent, with metal aqua complexes being the most extensively studied.^4^ The rate of this exchange depends on the nature of the metal ion, particularly ionic radii, charge, and coordination environment, ranging from 200 ps for Cs^+^ to 300 years for Ir^3+^.^4^ Coordination with nonaqueous solvents such as alcohols, dimethyl sulfoxide (DMSO), acetonitrile (MeCN), and amides, has also been explored.^5^

Among the cations investigated, significant efforts have been made to study Mg^2+^ complexes due to their prominent role in biology, including RNA folding, ATP hydrolysis, cellular signalling, and photosynthesis.^6^ In aqueous solution, Mg^2+^ forms octahedral [Mg(H_2_O)_6_]^2+^ complexes with a Mg–O distance of 2.10 Å, surrounded by a second solvation shell of 12 water molecules.^7–9^ Water molecules in the first solvation shell are tightly bound to the cation and undergo exchange with the bulk solvent molecules on the microsecond timescale (*k* = 5.3 × 10^5^ s^−1^ at 298 K) *via* a dissociative or interchange–dissociative mechanism.^10,11^

Another important example is Pd^2+^, which although less prevalent in biology has an irreplaceable role in organocatalysis^12–14^ and supramolecular chemistry.^15–20^ Pd^2+^ complexes have a square planar geometry defined by four coordinate bonds in equatorial positions. The complex can additionally interact with two more loosely bound ligands at the axial positions. In water, the [Pd(H_2_O)_4_]^2+^ complex has a Pd–O equatorial bond distance of 2.00–2.05 Å, with a second solvation shell of 10 waters located between 4.02–4.40 Å.^21,22^ The Pd–O axial distance has been investigated by neutron diffraction^23^ and extended X-ray absorption fine structure experiments^24^ and with different computational methods.^21,25,26^ The axial interaction distance is reported to range from 2.5 to 3.0 Å. Ligand exchange in Pd^2+^ square planar complexes is suggested to occur *via* an associative mechanism involving a pentacoordinated trigonal bipyramidal transition state (TS), as suggested by ligand field theory^27,28^ and supported by DFT calculations.^29^

While Mg^2+^ has a prominent role in biology, Pd^2+^ is a key building block in supramolecular chemistry, giving rise to a wide range of metallocages of various sizes and shapes.^30–33^ The interplay between the metal, organic ligands, and solvents determines the final assembled structure.^34–36^ Notably, the labile nature of Pd^2+^-ligand axial interactions is key for self-correction and optimal self-assembly.^37,38^ Pd^2+^-based metal–organic cages are commonly formed in MeCN solvent,^34,37,38^ although water and DMSO are also widely-used. [Pd(MeCN)_4_]^2+^ has been characterised using single crystal X-ray diffraction, revealing a Pd–N bond length of 1.956 ± 0.008 Å.^39^ NMR studies have explored MeCN ligand exchange, reporting reaction rates of *k* = 4.0 s^−1^ and *k* = 3.5 s^−1^ at 322 K.^40,41^

Computational modelling of Mg^2+^ and Pd^2+^ cations has received significant attention, in particular for the former. Approaches employed for their modelling include molecular dynamics (MD) simulations with classical force fields (FFs),^21,42^ quantum mechanics/molecular mechanics (QM/MM) methods,^25,43–45^ and *ab initio* MD (AIMD).^9,22,26,46–48^ MD simulations with non-polarizable FFs are widely use, aiming to balance cost and accuracy. Here, the metal ions are modelled as single or a small set of point charges with the electrostatic, dispersion, and excluded volume interactions taken into account by pairwise interaction potentials. The Lennard-Jones (LJ) parameters, and charges if a dummy model is used, are typically adjusted to reproduce experimental solution properties such as solvation free energy, coordination number, and water–metal distance of the first hydration shell, and, in some cases, the rate of water exchange.^49–51^ A 12-6-4 LJ potential has been developed to partially account for charge-induced dipole interactions *via* the *r*^−4^ term.^52,53^ However, none of the available Mg^2+^ FF parameters can simultaneously reproduce all properties with high accuracy. Moreover, given the focus on metal–water properties alone, these models cannot describe orbital-specific and anisotropic features important in many metal-containing protein or synthetic catalyst active sites, or even the properties of simple electrolytes.^54^ Polarizable FFs are in principle able to remediate the limitations of non-polarizable FFs, but they are less frequently used due to their time-consuming parameterization and increased computational cost, especially for exploring long-timescale processes.^42,50^ Lemkul and MacKerell^55^ modified Mg^2+^ parameters to describe its interaction with water, Cl^−^ ions, and nucleic acids using a polarizable FF based on the Drude oscillator model. This approach uses QM-computed interaction energies and geometries of hydrated complexes as a reference as well as condensed-phase osmotic pressure calculations. Mg^2+^ parameters for the AMOEBA force field were reported by Jiao *et al.*^56^ and further refined by Piquemal *et al.*^57^ However, both implementations experienced rapid water dissociation. This issue was addressed by Kurnikov and Kurnikova,^58^ who introduced a distance-dependent polarization response for water.

The effect of various FFs on the ligand exchange mechanism in the Mg[(H_2_O)_6_]^2+^ complex was extensively studied by Schwierz and colleagues, using transition path sampling.^59,60^ They demonstrated that while the commonly used non-polarizable FFs correctly predict the dissociative characteristics of the mechanism of the water exchange (*I*_d_), they tend to overestimate the free energy barrier, leading to a significantly slower reaction rate.^59^ In comparison, the polarizable FF Amoeba and specialized non-polarizable FF *MicroMg*, lead to a preference for an associative mechanism (*I*_a_) with a too low reaction barrier, leading to significantly faster reaction rates, further illustrating the complexity of accurately modelling the ligand exchange process.^60^

The interactions of Pd^2+^ with water molecules have also been studied computationally with classical FF approaches. Sanchez Marcos *et al.*^21^ investigated the [Pd(H_2_O)_4_]^2+^ complex in water using MD simulations. They developed two intermolecular potentials to describe the interactions between Pd^2+^ and the water molecules, one for the first solvation shell, fitted to interaction energies computed at the MP2 level on the gas-phase complex, and another for the hydrated ion-bulk water interactions by incorporating a continuum polarizable model to account for solvation effects.^21^ They suggested the presence of solvent molecules in the axial position located between 2.5 and 3.0 Å, referred to as a ‘mesoshell’. The concept of the mesoshell has sparked debate within the scientific community, with recent studies suggesting that the structure of Pd^2+^ aqua complexes in water should be interpreted under the ‘extended first shell’ paradigm.^25,44^ For example, utilising QM/MM methods, Adnan Ali Shah *et al.*^25,44^ identified a weakly bound axial ligand (Pd–O distance of 2.8 Å), resulting in a broad peak in the radial distribution function (RDF) between the first and second solvation shells. Contrasting findings were reported by Chen *et al.*^26^ using subsystem DFT AIMD simulations of the same complex. Their results indicated that water molecules rarely occupied the axial region. Instead, solvent molecules formed a protective “dome” on both sides of the square planar complex *via* strong hydrogen bonds, preventing the penetration of single water molecules from the axial direction. These studies provide alternative interpretations of the experimental data, underscoring the complex nature of axial interactions in Pd^2+^ aqua complexes.

AIMD simulations of explicitly solvated metal cations could, in principle, provide unbiased insights into the structural properties of the solvation shells and mechanisms of the ligand exchange by describing the entire system at the QM level, thus overcoming the limitations of classical FFs and QM/MM methods.^22,26,46,61–63^ However, its high computational cost limits its use to small systems and picosecond timescale processes, often insufficient to obtain converged free energies and model ligand-exchange processes. Machine learning potentials (MLPs) have emerged as promising alternatives to AIMD, reproducing accurate energies and forces from electronic structure reference calculations at a much lower cost.^64^ MLPs have been extensively used in modelling materials,^65,66^ organic molecules,^67,68^ and more recently in chemical reactivity.^69,70^ However, their extension to metal ions in solution remains less explored. Only a handful of examples have been recently reported, including the work of Liu *et al.*^71^ who employed DeepMD^72,73^ to model Mg^2+^ and Ca^2+^ in water in the presence of hydroxide. Mondal *et al.*, used DeepMD to study different formation and dissociation reactions in alkali carbonate–hydroxide electrolytes.^74^ Additionally, Michaelides *et al.* utilised the Behler–Parrinello Neural Network Potentials (NNPs)^75^ to model Na–Cl ion-paring in aqueous solution^76^ and in electrolytes confined to nanoscale pores.^77^

Traditionally, MLPs are trained using AIMD reference data under periodic boundary conditions (PBC). This approach inherently captures long-range interactions but incurs a high computational cost due to the large size of the system, primarily consisting of solvent molecules. Consequently, this limits the use of high levels of theory and restricts the achievable sampling. Previous work by our group^78,79^ and others^80,81^ have demonstrated the efficiency and accuracy achievable using cluster data for training. When combined with active learning (AL), which iteratively builds the training set based on a preliminary version of the trained MLP, this approach yields accurate and data-efficient MLPs at a low computational cost. In this study, we expand this protocol to model metal complexes in solution, using clusters of solvated metal ions for training. Specifically, we apply atomic cluster expansion (ACE)^82^ and its message-passing neural network-based variant (MACE)^83^ to two model systems: Mg^2+^ complexes in aqueous solution, representing a strongly interacting and biologically relevant metal ion; and Pd^2+^ complexes in acetonitrile (MeCN), a transition metal relevant for supramolecular chemistry in non-aqueous solvents.^34,37,38^ Ligand exchange in these complexes proceeds *via* different mechanisms, allowing us to investigate the capability of the MACE potentials to model structural and energetic features characteristic of both processes.

## 2 Methods

### 2.1 ACE and MACE machine-learning potentials

In this work, MLPs were trained using linear regression with the ACE^84^ descriptor and its variant MACE, in which ACE is combined with an equivariant message-passing neural network architecture.^83^

The ACE descriptor^85^ builds on a traditional many-body expansion, where the potential energy surface (PES) of the system is expressed as a sum of different body-order interactions, including two-body, three-body, and higher-order interactions depending on the truncation. Although this approach is physically motivated, it is limited to modestly-sized molecular systems, as the computational cost of evaluating the energy exponentially scales with the system size, making it impractical to consider interactions beyond the three-body order. ACE overcomes this limitation by introducing the concept of atomic neighbour density, where the energy of each atom depends on the many-body interactions with its N neighbours within a defined cut-off radius. The validity of this concept is based on the assumption that the energy of each atom only depends on its local environment.^75,86^ Second, it projects these densities onto physically invariant basis functions. This procedure ensures that the evaluation cost of many-body terms scales linearly, rather than exponentially, with the number of neighbours, regardless of the body order. A detailed description of the method is provided in ref. 85. The capability of the ACE descriptor to accurately map the PES enables the use of simple linear regression for fitting, resulting in an accurate and data-efficient approach to train MLPs.^84^

MACE combines the ACE descriptor with an equivariant message-passing neural network architecture,^83^ incorporating body-order contributions as node features. Using graph neural networks (GNNs), the body-order term and cluster region implicitly expand with the number of message-passing layers, resulting in a more accurate representation of atomic environments.^83^

Both ACE and MACE have been shown to reliably predict the energies and forces of molecules and condensed phase systems.^84,87–89^ Linear ACE provides high accuracy in low-data regimes, making it particularly suitable for use in the early stages of AL, where typically small data sets are used.^78,79^ In this work, we use linear ACE to build the training data sets using the AL loop, while MACE is used to expand the data sets and fine-tune the final potentials used for production of MD simulations.

### 2.2 Workflow

**Fig. 1:**
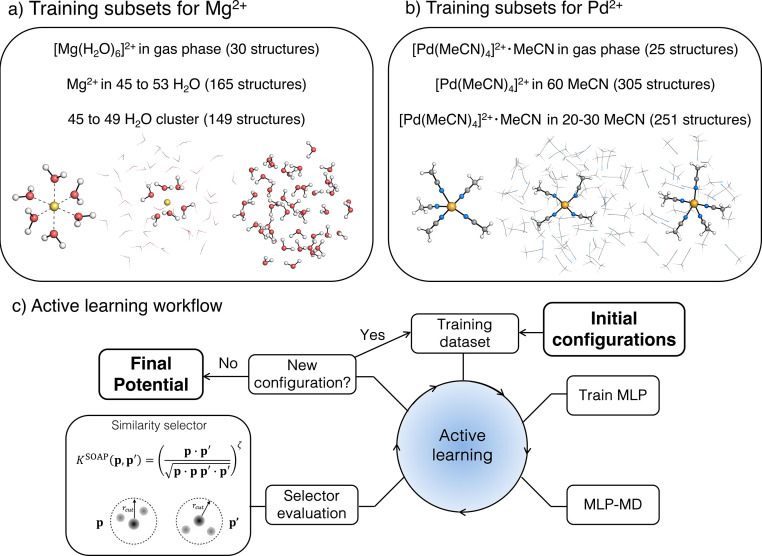
Training data and active learning workflow: (a) training subsets for Mg^2+^ in aqueous solution, (b) training subset for Pd^2+^ in acetonitrile (MeCN), (c) scheme of the active learning workflow used to train the machine-learning potentials (MLPs).

The workflow presented here builds on our previous work on an automated AL strategy for modelling chemical reactions in explicit solvents (Fig. 1).^79^ The AL cycle is initiated from a small training set of approximately 10 structures. These data consist of gas-phase molecules generated by random displacement from a QM-optimized structure or solvated clusters, obtained from MD simulations or random placement of molecules in a box. The structures are labelled with energies and forces computed at the reference level of theory. The initial training data set is extended using AL as follows: a first version of the MLP is generated and used to propagate several independent MD simulations, typically ten, for *n*^3^ + 2 fs, where *n* is the index of the MD run in the AL loop, starting from 0. From these trajectories, new structures are selected using a similarity selector,^79^ which identifies new structures to be included in the training based on the similarity between a global smooth overlap of atomic positions (SOAP) representation of data point *p* and configurations *p*′ in the existing training set.^90^ The similarity vector, ***K***, is defined as follows:

1 ***K*** = (|*k*(***p***_0_·***p***_i_)|^*ζ*^, |*k*(***p***_0_·***p***_j_)|^*ζ*^,⋯)^*T*^

where the components of the vector *k*(*p*·*p*′) correspond to the SOAP kernel functions computed between the SOAP representation of the new structure ***p***_0_ and the *i*-th configuration in the existing training data ***p***_i_. Parameter *ζ* is a positive integer that increases the sensitivity of the kernel to changes in atomic position.^90^ The selector adds structures to the training set if the maximum value of their similarity vector, ***K***, is smaller than the given threshold *k*_T_, *i.e.*, max(***K***) < *k*_T_. The new structures are then labelled by the reference energy and forces, added to the training set and the potential is retrained. If no structures are selected from the trajectories, the index *n* increases by one and a longer MD simulation is performed with the same potential. The AL procedure is repeated until it either reaches the maximum number of AL cycles or when no new structure is selected within the maximum AL time. Details on electronic structure and MD protocols are provided in the Computational details section.

### 2.3 Data set preparation

#### 2.3.1 Mg^2+^ in water

The training data set for Mg^2+^ consists of 344 structures. To increase the accuracy of the resulting potentials across systems with different sizes, the final dataset combines three subsets with different compositions (Fig. 1a): (i) [Mg(H_2_O)_6_]^2+^ complex in the gas phase (30 structures), (ii) Mg^2+^ solvated in 45 to 53 water molecules in a spherical cluster with radius 7 Å (165 structures), and (iii) water clusters containing 45 to 49 water molecules placed in a spherical cluster with radius 7 Å (149 structures). The cluster size was selected to be larger than the 6 Å distance cut-off of the descriptors used in MLP, needed to cover the Mg–O distance range sampled in the dissociative mechanism. All datasets were trained using the energies and forces computed at ωB97X-D3BJ/def2-TZVP^91,92^ level of theory as ground truth, which provides accurate estimation of structural and thermodynamic properties of large systems. The ACE MLP was used to generate the structures during AL unless specified otherwise.

##### 2.3.1.1 [Mg(H_2_O)_6_]^2+^ complex in the gas phase

AL was initiated from 10 structures obtained by a random displacement of atoms in [Mg(H_2_O)_6_]^2+^ complex in the gas phase. The new structures were selected using the similarity selector with a SOAP cut-off of 5 Å and threshold of 0.999, with the maximum time in the active training loop set to 3 ps. This procedure led to the selection of 30 structures.

##### 2.3.1.2 Water cluster subset

The initial structures of the bulk water system were prepared by classical MD simulation using TIP4P-Ew FF.^93^ A cubic box (*L* = 15 Å) was solvated with 112 water molecules, minimized and equilibrated in an *NPT* ensemble (300 K and 1.0 bar) for 1 ns using Langevin dynamics and the Berendsen barostat as implemented in the sander module of Ambertools23.^94^ For the initial training set, 10 spherical clusters of 7 Å radius containing 45 to 49 water molecules were extracted from the equilibrated trajectory and labelled with the reference energies and forces (*vide infra*). The training set was then enhanced by AL using the similarity selector with a threshold of 0.9998 to avoid selection of structures that are too distorted, and a maximum time set to 10 ps to accommodate water relaxation. This approach yielded a total of 149 structures.

##### 2.3.1.3 Mg^2+^ complex in a water cluster

The initial structures for the training were generated using classical MD simulations including a Mg^2+^ ion solvated by 325 water molecules in a box of 21.5 Å. The box was minimized and equilibrated to experimental water density using the same procedure as pure water. The system was modelled using the TIP4P-Ew water model combined with Li/Merz ion parameters in TIP4P-Ew water (12-6 HFE set).^52^ 10 structures were randomly extracted from the equilibration trajectory and cut into a 7 Å sphere centred at the Mg^2+^ cation.

The three subsets were combined to form the full data set used to initiate AL, which was performed in four phases. In the first phase, the ACE potential was trained on the full data set and used in AL with MLP-MD initiated from the Mg^2+^-water cluster (structure selected from a subset (ii)). During the MLP-MD dynamics step, the cluster was constrained in its spherical shape by a flat-bottom spherical bias potential set at a distance of 8 Å from the Mg^2+^ cation, applying a harmonic restraint of 500 kcal mol^−1^ Å^−2^. MD simulations during AL were performed in an NVT ensemble at 400 K. New structures were selected using the similarity selector with a threshold of 0.999, with maximum time in AL set to 7 ps. This longer AL time compared to the gas phase complex was used to account for the higher flexibility of water molecules outside the first solvation shell. The first phase yielded 11 structures, which were added to the data set.

In the second phase, the AL was initiated using one of the clusters generated during the previous phase, with the radius reduced by placing the harmonic bias at a distance of 7.5 Å to increase the density of the solvent around Mg^2+^ and enhance the sampling of the repulsive region of the potential. To avoid selecting overly distorted structures resulting from potentially unstable dynamics, the selector threshold was tightened to 0.9999. Simultaneously, the AL time was increased to 20 ps to further capture longer water dynamics. The second phase resulted in generating an additional 55 structures.

After these two phases of training, we tested the accuracy and stability of the resulting potential by comparing the MLP energies and forces with the ground truth data (for details see ESI S2†) and conservation of the total energy in NVE dynamics. Despite the high accuracy of the trained ACE potential, as evidenced by a mean absolute deviation (MAD) of 0.79 meV per atom for energy and 46 meV Å^−1^ for forces (Fig. S1†), the NVE and NVT MD simulations using PBC with the ACE potential were unstable. This included the formation of vacuum bubbles inside the solvent, followed by the system's collapse. These instabilities could be alleviated by introducing more radial functions into the descriptor and tuning the hyperparameters. However, we decided to change the model in the following phases from ACE to MACE, which is computationally more efficient. Indeed, the MACE potential provided stable NVE dynamics under PBC using the same training data set. Interestingly, the long NVE dynamics with the MACE potential promoted proton transfer of a water molecule in the first solvation shell, leading to the formation of [Mg(H_2_O)_5_(OH)]^+^ and H_3_O^+^ species. As these structures were underrepresented in the previous AL loops, resulting in larger prediction errors (see Fig. S2†), we manually selected 35 structures along the *NVE* trajectory, cut them into 7 Å clusters, which contained the species, and added them to the training set for the third training phase. Apart from adding these data, we further repeated the AL loop to account for possible differences between the conformational space sampled by ACE and MACE potentials. The re-trained MACE potential was again found to provide an accurate estimate of energies and forces, 0.69 meV per atom and 29 meV Å^−1^ (see Fig. S3†). The preliminary NVT dynamics with this potential, however, led to fast dissociation of one H_2_O molecule from the first solvation shell, with no exchange with the bulk solvent. To correct for this behaviour and to ensure accurate exchange of the water molecules around the Mg^2+^, we completed the training set by adding 23 structures with the H_2_O molecule dissociated to a distance over 3.0 Å (see Fig. S4†) from Mg^2+^ within the fourth training phase.

#### 2.3.2 Pd^2+^ in MeCN

The MLP for the Pd^2+^ complex was trained using a total of 581 data points from the following subsets (Fig. 1b). (i) Data obtained by a relaxed 2D scan of the [Pd(MeCN)_4_]^2+^·MeCN complex in the gas phase along the two Pd–N bonds describing the ligand exchange process (25 structures). (ii) [Pd(MeCN)_4_]^2+^ complex solvated by 60 MeCN molecules (305 structures). (iii) The [Pd(MeCN)_4_]^2+^·MeCN complex solvated by 20 to 30 MeCN molecules to describe interactions between Pd and MeCN (251 structures). As in the Mg^2+^ case, the size of all clusters was selected in a way that the resulting cluster radius exceeds the 6 Å cut-off used in ACE and MACE descriptors. Unless specified otherwise, ACE was used as the ML model in all training phases, employing the ground truth potential TPSS0-D3BJ/def2-TZVP,^92,95,96^ since this functional has shown good performance in reactions involving late transition metals.^95^ MD simulations in the AL loops were performed in an NVT ensemble at 300 K. MD simulations longer than 1 ps used a flat-bottom spherical harmonic bias potential set at a distance from the Pd^2+^ cation, applying a harmonic restraint of 100 kcal mol^−1^ Å^−2^. The value of the harmonic restraint has been chosen to maintain the integrity of the cluster without creating artefacts from the pulling force. The onset distance of the flat-bottom potential was varied according to the size of the clusters so that the density of the cluster was close to the experimental density of the (bulk) liquid.

##### 2.3.2.1 [Pd(MeCN)_4_]^2+^·MeCN complex in the gas phase

The transition state (TS) structure corresponding to pentacoordinated trigonal bipyramid was obtained at the TPSS0-D3BJ/def2-TZVP level of theory (Fig. 1b) and was used as a starting point for a 2D relaxed PES scan along the two Pd–N bonds involved in the ligand exchange. These bonds are of equal length in the TS (2.28 Å); while the length of the remaining three Pd–N bonds is 1.95 Å. The PES scan resulted in a total of 25 structures (5 × 5 grid). In the following paragraphs, TS refers to the transition state structure of [Pd(MeCN)_4_]^2+^·MeCN obtained from the PES scan mentioned above, while reactant state (RS) refers to the structure obtained from the geometry optimization of the TS in the gas-phase with TPSS0-D3BJ/def2-TZVP.

##### 2.3.2.2 [Pd(MeCN)_4_]^2+^ complex solvated in clusters of 60 MeCN molecules

The initial structure for the subset was generated with the quantum cluster growth (QCG) method^97^ as implemented in xTB (version 6.5.0)^98^ and the lowest energy conformer was selected with CREST.^99^ Then, the conformational space was explored *via* metadynamics simulations with xTB.^98,100^ Three simulations of 25 ps each were run in the NVT ensemble at 600 K, using the Cartesian root-mean-square-deviation (RMSD), with respect to a list of reference structures updated every 2 ps, as a collective variable.^100^ During the simulations, a flat-bottom spherical bias potential was applied to keep the cluster at the experimental density of liquid acetonitrile at room temperature.^100^ For each of the three trajectories, the first 600 fs were excluded and the remaining frames were merged in a single trajectory. From this, frames have been extracted every 240 fs resulting in 305 structures.

##### 2.3.2.3 [Pd(MeCN)_4_]^2+^·MeCN complex in clusters of 20–30 MeCN molecules

The structures used as starting conformations in the multi-step AL approach described in this section have been generated with CREST. The TS and RS structures obtained in the gas phase (from the dataset (i)) were solvated by 20 to 30 MeCN molecules using the QCG method^97^ and the lowest energy conformation of the cluster was selected with CREST,^99^ as mentioned above. Different conformations were generated for the different AL training phases as follows. Firstly, the data set (i) was used as an initial data set in the AL loop, starting from TS solvated by 20 MeCN molecules; new structures were selected using the similarity selector with a threshold of 0.9999, with a maximum MD simulation time set to 750 fs, the time found to be required for transitioning from trigonal bipyramidal to square-pyramidal coordination geometries, observed from previous trials. This AL phase yielded 56 structures.

In the second phase, dataset (i) and the 56 structures obtained from the first phase were employed as the starting training set. The TS solvated by 30 MeCN molecules was used as a starting conformation for the new AL loop, with an MD simulation time of 1.5 ps and a SOAP selector threshold of 0.999. The choices to extend the MD simulation time and the number of solvating MeCN molecules were made to capture how the relaxation from the transition state would evolve on longer timescales and in the context of a larger solvation environment. The selector threshold was relaxed to avoid the selection of conformations too similar to the starting one since the fluctuations along the TS relaxation have been explored in the previous training phase. In this phase, 36 new structures were generated, expanding the dataset to 117 structures.

In the third phase, the RS solvated by 20 MeCN molecules was used as a starting structure, with the AL MD time extended to 5 ps to allow sampling of more distant regions of the PES. Therefore, the number of solvent molecules was decreased to lower the computational cost. Thirty new structures were generated and added to the previous data, yielding a total of 147 data points. Preliminary validation in the NVT ensemble under PBC showed artefacts in the description of the average structure of the system, in particular, the formation of void regions in the axial positions of the complex (Fig. S5†).

In line with the Mg^2+^ case, we, therefore, decided to adopt the MACE for AL due to its computational efficiency and accuracy.^89^ Furthermore, to enhance the stability of the potential during AL, we selected 150 structures from the dataset (ii) and merged them with the previous 147 structures, obtaining an extended starting dataset. A final AL loop was started from RS solvated by 20 MeCN molecules, using a similarity selector threshold of 0.9999 and simulation time set to 20 ps to ensure the stability of the potential on longer timescales. This training phase resulted in 129 new structures. Eventually, not considering the 25 structures from dataset (i), the multi-step AL approach described above yielded a total of 251 structures (147 + 129 − 25).

## 3 Results and discussion

### 3.1 Validation of the MLP

As a first step, we validated the accuracy and stability of the generated MACE potentials for both Mg^2+^ in aqueous solution and Pd^2+^ in MeCN. To evaluate the prediction error on unseen data, we generated an ensemble of testing structures using an MLP-MD simulation of the metal ion in a spherical cluster of solvent molecules. We then selected frames along the trajectory and performed a point-to-point comparison between the energies and forces computed at the ground truth DFT level of theory and MACE.

**Fig. 2:**
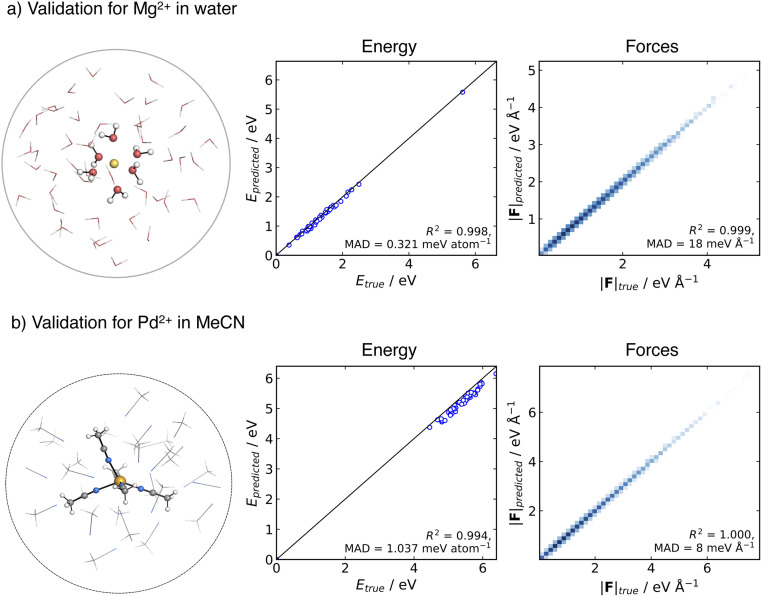
Comparison of the ground-truth and MACE prediction of energies and forces for cluster systems at 300 K: (a) Mg^2+^ solvated in 51 water molecules modelled at ωB97X-D3BJ/def2-TZVP level of theory, (b) Pd^2+^ solvated in 30 MeCN molecules modelled at TPSS0-D3BJ/def2-TZVP level of theory.

The testing data set for the Mg^2+^ cation in aqueous solution consisted of 51 structures collected over 50 ps NVT dynamics of Mg^2+^ solvated in a cluster of 51 water molecules. The spherical shape of the cluster was kept using a harmonic spherical potential placed at 7.5 Å from Mg^2+^. Validation results are depicted in Fig. 2a. MACE demonstrates excellent performance in energies and forces, with an MAD of 0.31 meV per atom and 18 meV Å^−1^ for energies and forces, respectively.

For Pd^2+^ in acetonitrile, the MACE potential was tested on structures generated from 100 ps NVT dynamics using a cluster containing Pd^2+^ and 30 MeCN molecules. The solvent molecules in the cluster were confined by a flat-bottom spherical bias potential with a radius of 10.0 Å, centred on Pd^2+^. From the trajectory, 51 structures were extracted. The MAD is 1.04 meV per atom and 8 meV Å^−1^ for energy and forces, respectively. The good correlation with respect to the ground truth energies and the accuracy in forces suggest that the MACE potential closely reproduces the shape of the reference PES, with the higher energy MAD likely arising from a systematic shift in the absolute values, which has been previously reported in some instances with MACE.^89^ Overall, the MACE potentials provide a reliable prediction of energies and forces for both tested systems.

To further assess the performance of the MACE potentials in larger systems, we performed 100 ps MD simulations under PBC in the NVE ensemble for a system consisting of Mg^2+^ with 145 water molecules in a 16.3 Å box and Pd^2+^ with 159 MeCN molecules in a 24.0 Å box. In both cases, the MACE potential conserved energy, confirming the stability of the dynamics under PBC and on simulation times longer than the active learning time (Fig. S7 and S8†).

### 3.2 Structural properties of the metal solvation shells

**Fig. 3:**
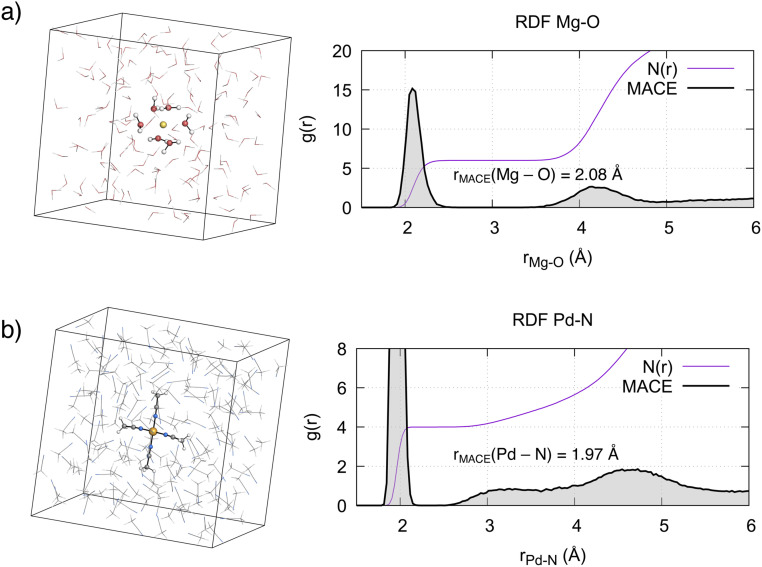
Simulation boxes, radial distribution functions *g*(*r*) and coordination numbers *N*(*r*) of the metal complexes in solution. (a) Mg^2+^ in aqueous solution, (b) Pd^2+^ in MeCN.

We evaluated the structural properties of the metal environment by computing the radial distribution functions (RDFs) between the metal ions and the coordinating solvent atom. For [Mg(H_2_O)_6_]^2+^, the computed Mg–O RDF from 500 ps MD simulations (Fig. 3a) shows a first peak at 2.08 Å, in agreement with the 2.10 Å reported from X-ray diffraction and neutron scattering experiments.^7,101^ Integration of this curve results in a coordination number of 6, confirming the octahedral arrangement of this complex. A second, less well-defined peak is evident around *r* = 4.15 Å, corresponding to the second solvation shell. Integration of this peak results in a coordination number of 12, in agreement with experiments^7^ and reported *ab initio* computations.^9^ In line with the known lifetime of the first solvation shell, the octahedral complex remained stable during the simulation time and no ligand exchange with the bulk solvent was observed.

As expected from ligand field theory,^27^ Pd^2+^ forms a square planar complex with 4 MeCN molecules, with a Pd–N distance of 1.97 Å (Fig. 3b). This is in excellent agreement with the value obtained from the single crystal X-ray diffraction (SC-XRD) of the complex (Pd–N bond 1.96 ± 0.01 Å).^39^ Two peaks, one around 3.3 Å, with a shoulder starting from 2.5 Å, and another around 4.6 Å, are also observed. The latter peak is associated with the second solvation shell, with a coordination number of 8. The former peak corresponds to the interactions of acetonitrile molecules in the axial position, with a coordination number increasing from 4 to 6. The previous studies on Pd^2+^ aqua complexes provided a foundation for understanding the axial interactions of Pd^2+^ with MeCN. As mentioned in the introduction, two paradigms exist in the literature. In the mesoshell paradigm, the two axial ligands are symmetrically bound, resulting in a sharp peak between the first and second solvation shells.^21,23,24^ Conversely, the “extended first solvation shell” concept suggests more weakly bound axial ligands, leading to the presence of a broad peak.^25,44^ The structural features observed in the RDF in Fig. 3b indicate that for Pd^2+^ in acetonitrile, axial ligands interact according to the “extended first solvation shell” paradigm. This notion is further supported by the asymmetry in the average distance of the two axial MeCN ligands with respect to Pd^2+^ (Fig. S9 and S10†). Additionally, a detailed analysis of the axial coordination pattern (see Section S3.1†) suggests that the preferred average structure of the complex is not octahedral but square pyramidal, with the ratio between the latter and the former being 7 : 4.

### 3.3 Free energy barrier of ligand exchange

The ability of the MACE potentials to describe ligand exchange around the metal centre relies on accurately describing the different coordination states and their exchange mechanisms, ideally leading to accurate kinetics. The latter has been difficult to achieve with classical force fields.^59,60^

**Fig. 4:**
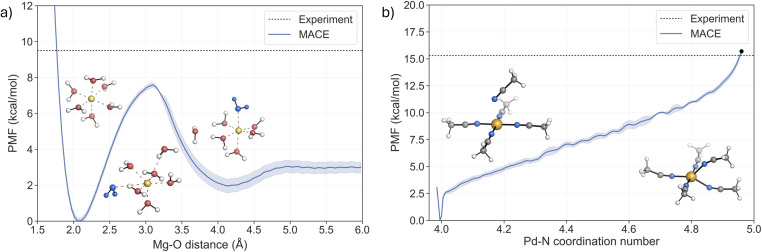
Potential of mean force (PMF) profiles of the two ligand exchange processes for (a) [Mg(H_2_O)_6_]^2+^ (the solvent molecule exchanged is coloured blue) and (b) [Pd(MeCN)_4_]^2+^, where the black dot indicates the energy at the TS.

As discussed previously, ligand exchange in Mg^2+^ complexes is suggested to follow a dissociative or interchange–dissociative mechanism, which proceeds through an intermediate or transient structure with a lower coordination number. Solvent dissociation from the first solvation shell of [Mg(H_2_O)_6_]^2+^ thus represents the rate-limiting step of the process. The free energy barrier associated with the dissociation of one solvent molecule was obtained from the potential of mean force (PMF) using US with 48 windows (Fig. 4a). The PMF shows a minimum at 2.1 Å, in agreement with the value obtained from the RDF (Fig. 3a). A second shallow minimum is located at around 4.25 Å, corresponding to the position of the second solvation shell, which is approximately 2 kcal mol^−1^ higher than the first minimum. This indicates that the sampling of the region where water leaves the first solvation shell is not fully converged. However, the two repetitions of the US confirm that the free energy barrier is not affected (see Fig. S13†). The representative structures of both minima and the associated transition region are depicted in Fig. 4. Analysis of the trajectories confirms that the MACE potential correctly restores the octahedral geometry of the complex. The barrier of pulling the water molecule away from the first solvation shell is 7.6 ± 0.15 kcal mol^−1^, with the peak located at a distance around 3.1 Å. The predicted barrier is 1.9 kcal mol^−1^ lower than the experimental value, 9.5 kcal mol^−1^,^4,11^ implying a higher rate of water exchange around the Mg^2+^.

For the Pd^2+^ complex with MeCN, an associative mechanism has been suggested from NMR experiments and static electronic structure calculations.^27–29,40,41^ To determine the coordination number that corresponds to the TS, the fluctuations of the coordination number and the Pd–N bond lengths were analysed in the umbrella sampling trajectory that corresponds to the TS region in the PMF (Fig. S16†). The coordination number fluctuates around a mean value of 4.96, which yields a barrier of 15.7 kcal mol^−1^ in the PMF (black dot in Fig. 4b). This value is in very good agreement with the experimental values from two independent NMR studies, 15.3 ± 0.4 kcal mol^−1^ (ref. 40) and 15.2 ± 0.2 kcal mol^−1^.^41^

The reaction mechanism of the ligand exchange is illustrated in detail by the representative snapshots in Fig. 4b. The solvent association proceeds *via* the formation of a square pyramid, where the axial Pd–N bond shortens as the system approaches the TS region, while one of the equatorial Pd–N bonds progressively elongates. This process leads to the formation of a trigonal bipyramidal TS, with an equal length of two Pd–N bonds involved in the ligand exchange. Taken together, the PMF from the US simulations confirms the associative nature of the ligand exchange mechanism. Furthermore, the activation barrier is in excellent agreement with the experimental values, supporting the notion that the MACE potential fitted to the hybrid DFT reference accurately describes the PES of the system and allows for a realistic description of the dynamics of the ligand exchange process.

## 4 Conclusions

In this work, we present computational strategies to build training data sets for modelling ligand exchange processes of divalent metal cations in explicit solvents with MLPs. Using Mg^2+^ in aqueous solution and Pd^2+^ in MeCN as model systems and illustrative examples, we demonstrate the capability of the MACE potentials to reproduce the total energies and forces of the solvated metal cations. Furthermore, the MLPs trained on cluster data can be used in the condensed phase simulations with periodic boundary conditions. The MACE potentials yield metal ion–solvent RDFs in excellent agreement with experimental data, confirming the capacity of MACE to capture the structure of the polarised solvent shells around the cations. Moreover, we demonstrate the ability of the MACE potentials to model changes in the coordination shells of the metal cations, allowing for a structurally and energetically realistic description of different ligand exchange mechanisms in complex liquid environments. More generally, we show that the active learning strategy combined with MACE potentials allows the generation of accurate and data-efficient MLPs that are suitable to model changes in the coordination chemistry of charged species in solution. While further work is needed to automate the selection of accurate parameters suitable across different metals, this study provides a robust computational framework for preparing data-efficient models that accurately describe metal–ligand interactions, paving the way to modelling increasingly complex systems, such as metallocages and catalysts.

## 5 Computational details

### 5.1 Model parameters and training

ACE and MACE models were trained with ACE.jl (ref. 82) wrapped by pyjulip and mace v0.3.4 (ref. 83 and 102) using the in-house mlp-train package.^103^ The model hyperparameters and parameters used for the active learning (AL) cycles are listed in the ESI S1.† The QM computations were performed in ORCA v5.0.4 (ref. 104) wrapped with autodE.^105^ The reference energies and forces were computed at ωB97X-D3BJ/def2-TZVP ^91,92^ and TPSS0-D3BJ/def2-TZVP ^92,95,96^ levels of theory for the Mg^2+^ and Pd^2+^ systems, respectively.

### 5.2 Production MD

A Mg^2+^ cation in water was simulated in a periodic cubic box of 16.3 Å containing 1 cation and 145 water molecules. The initial structure for the dynamics was obtained by classical dynamics (see ESI S2.3.1†). The Pd^2+^ system was simulated in a periodic cubic box of 24.0 Å containing 1 metal atom and 159 acetonitrile molecules. The starting configuration of the system was built in three steps. Firstly, an NPT-equilibrated box of acetonitrile was obtained with the force field parameters from Caleman *et al.*^106^ Secondly, the xTB-optimized structure of the [Pd(MeCN)_4_]^2+^ complex was solvated with the acetonitrile box. Lastly, the whole system was energy-minimized with Gromacs (v2019.1).^107^ The radial distribution functions for both the Mg^2+^ and Pd^2+^ systems were computed from 500 ps NVT simulations with the MACE potentials, with the first 50 ps used for equilibration and skipped from the analysis. The equations of motion were integrated by the i-PI driver,^108^ with MACE potential evaluated by the MACE-Atomic Simulation Environment (ASE) calculator using ASE v3.23.0b1.^109^ All MD simulations were propagated with an integration time step of 0.5 fs. MD in the NVT ensemble was thermostatted at 300 K by a stochastic velocity rescaling thermostat with a coupling time constant of 100 fs.^110^

### 5.3 Free energy computations

The free energy profiles of the metal–ligand exchange reactions were evaluated using umbrella sampling (US) simulations using the i-PI driver combined with the Plumed v2.9.0 library.^111,112^ The PMFs were constructed using the Weighted Histogram Analysis Method (WHAM) code v2.0.11.^113^

For Mg^2+^ in water, the Mg⋯O distance was chosen as a reaction coordinate (RC). The starting structures for each window were generated by a steered MD, pulling a water molecule from the first solvation shell to a distance ranging from 1.5 to 7 Å. The US covered the distance from 1.5 to 6.0 Å, split into 48 windows with a spacing of 0.075 Å. In each window, the trajectory was propagated for 1 ns, with the first 50 ps used for equilibration and skipped from the analysis, corresponding to 45.6 ns of sampling time. The US windows were propagated independently at 300 K. A harmonic umbrella restraint with force constant 500 kcal mol^−1^ Å^−2^ was employed in the windows from 1.5 to 3.00 Å, while the force constant was lowered to 250 kcal mol^−1^ Å^−2^ in the region from 3.00 to 6.00 Å. The PMF was computed as an average from 2 US runs with random seeds for generating the initial velocity distributions, with an uncertainty estimated as a standard deviation. The final PMF was corrected by the entropy term 2*k*_B_*T* ln(*r*/*r*_0_) that accounts for the increasing volume of configuration space with increasing distance *r*.^114,115^

For Pd^2+^ in acetonitrile, the coordination number was chosen as a collective variable for the US (further details in Section ESI S4.2†). The starting structures for the US runs were generated by steered MD, during which one of the two MeCN axial ligands was pushed towards the Pd centre, guiding the system towards the ligand exchange event through the formation of the pentacoordinated TS. In the US, the coordination number was varied from 4 (square planar reactant state) to 5 (pentacoordinated TS, further details in ESI S4.2†). For each window, the simulation was run for 57.5 ps with a harmonic restraint with force constant 2400 kcal mol^−1^ Å^−2^, using the last 50 ps for the analysis. To estimate the statistical uncertainty, the US simulations were repeated three times (using different random seeds for generating the initial velocity distributions), and the standard deviation from these three repeats is plotted in Fig. 4.

## Data availability

Data for this article, including training datasets, along with the initial configurations of umbrella sampling trajectories, have been deposited in the Oxford Research Archive (ORA) at https://ora.ox.ac.uk/objects/uuid:da5912c9-3277-478c-a61d-ea2a8ccf588e. The code for the active learning and training of the MLPs can be found at https://github.com/duartegroup/mlp-train/tree/main/mlptrain with DOI https://doi.org/10.6084/m9.figshare.25816864.v1. The version of the code employed for this study is version v1.0.0b0.

## Author contributions

VJ, GT, LVS and FD conceptualised the study. VJ and GT performed the computations. All authors participated in data analyses and writing of the manuscript. VJ and GT wrote the first draft.

## Conflicts of interest

There are no conflicts to declare.

## Supplementary Material

FD-256-D4FD00140K-s001
