# Supplementary material for: Modelling ligand exchange in metal complexes with machine learning potentials
Source: Faraday Discuss. 2024 Aug 3;256:156–76. doi: 10.1039/d4fd00140k (PMC11417676; doi:10.1039/d4fd00140k)
Supplement: FD-256-D4FD00140K-s001 [file FD-256-D4FD00140K-s001.pdf]

Supplementary Information (SI) for Faraday Discussions.
This journal is © The Royal Society of Chemistry 2024

Supporting Information

Modelling ligand exchange in metal complexes with machine learning potentials

Veronika Juraskova, Gers Tusha, Hanwen Zhang, Lars V. Schäfer, Fernanda Duarte

Contents

S1 Hyperparameters **2**

S2 Training data set: Additional details **3**
S2.1 Validation of the ACE potential for Mg^{2+} system after the second AL phase 3
S2.2 Formation of $[Mg(H_2O)_5OH]^+ + H_3O^+$ 3
S2.3 Validation of the ACE potential for Mg^{2+} system after the third AL phase 4
S2.4 Prediction error on structures with dissociated H_2O from the first solvation shell . . 5
S2.5 Artefacts from the ACE potentials for the Pd^{2+} system 6
S2.6 Transferability of the MACE potential for Pd^{2+} in MeCN to simulating pure MeCN 7
S2.7 Stability in the NVE dynamics 8

S3 RDFs and structural information **9**
S3.1 Pd^{2+} in MeCN 9

S4 Umbrella Sampling simulations **10**
S4.1 Mg^{2+} system 10
S4.2 Pd^{2+} system 11

S1 Hyperparameters

Table S1 displays the hyperparameters used in the SOAP selector setting in the AL and in the training of ACE and MACE potentials.

Table S1: Hyperparameter setting for ACE and MACE potentials and SOAP descriptor. (* the polynomial degree for four- and five-body potentials do not include H)

Type	Parameter	Description	Value
ACE	ν	Maximum correlation order	4 (Mg^{2+}) / 3 (Pd^{2+})
	$D_{\nu}^{\max}$	Maximum polynomial degrees for corresponding body potential	20, 16, 16, 12 *
	r_{mb}	Outer cutoff radius for many-body potentials	6.0 Å
	r_{pair}	Cutoff radius for the pair potential	7.0 Å
MACE	ν	Maximum correlation order	3
	r_{mb}	Outer cutoff radius for many-body potentials	6.0 Å
	hidden_irreps	Irreducible representations	128x0e + 128x1o
	batch_size		5 (Mg^{2+}) / 4 (Pd^{2+})
	valid_fraction	Fraction of the data set used for validation during the training	0.1
	energy_weight		1.0
	forces_weight		5.0
SOAP descriptor	$\sigma_{\mathrm{at}}^{\mathrm{SOAP}}$	Spread of the Gaussian added to atomic density	1.0 Å
	$n_{\max}, l_{\max}$	The maximum number and degree for the radial (n) and angular (l) basis	6
	r_{cut}	Cutoff distance for local region	5.0 Å

S2 Training data set: Additional details

S2.1 Validation of the ACE potential for Mg^{2+} system after the second AL phase

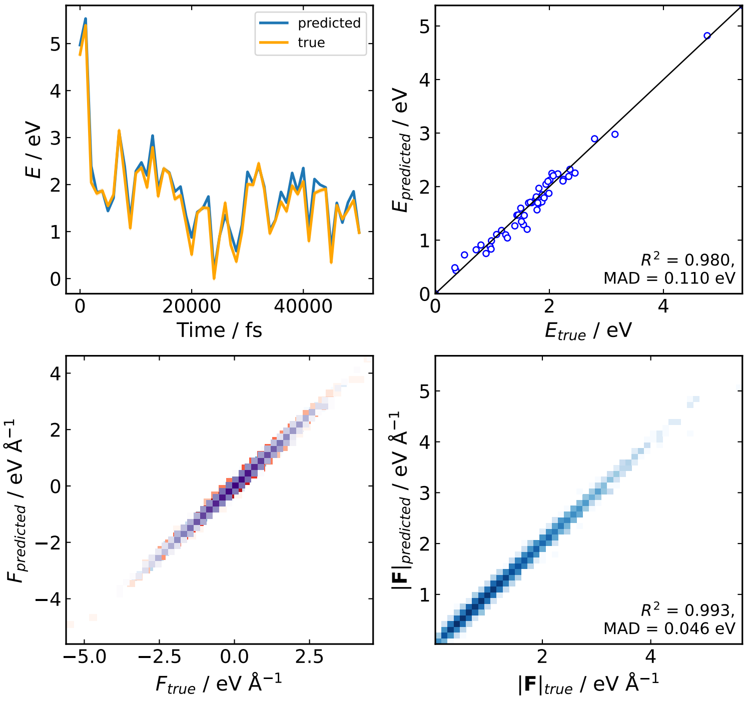

Figure S1: Comparison of ground-truth (ωB97X-D3BJ/def2-TZVP) and predicted (ACE MLP, trained after second phase of AL) energies and forces over 50 ps trajectory of the cluster containing Mg^{2+} in 46 H_2O molecules.

S2.2 Formation of $[Mg(H_2O)_5OH]^+ + H_3O^+$

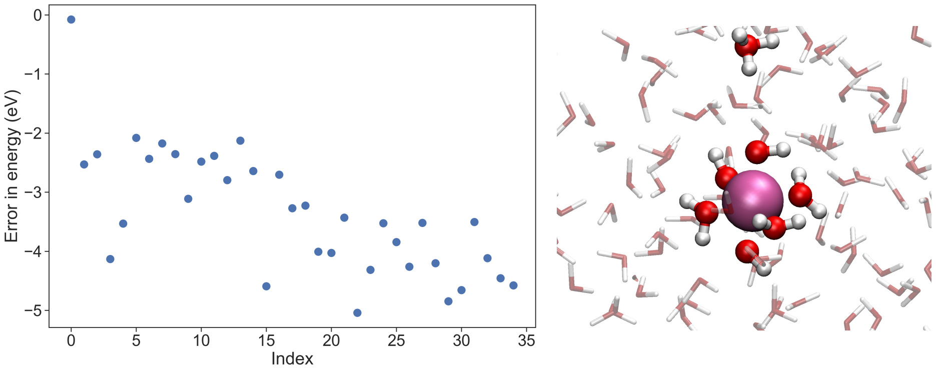

Figure S2: Energy error prediction of structures containing $[Mg(H_2O)_5OH]^+$ and H_3O^+ species and illustrative snapshot of the structure in the data set.

S2.3 Validation of the ACE potential for Mg^{2+} system after the third AL phase

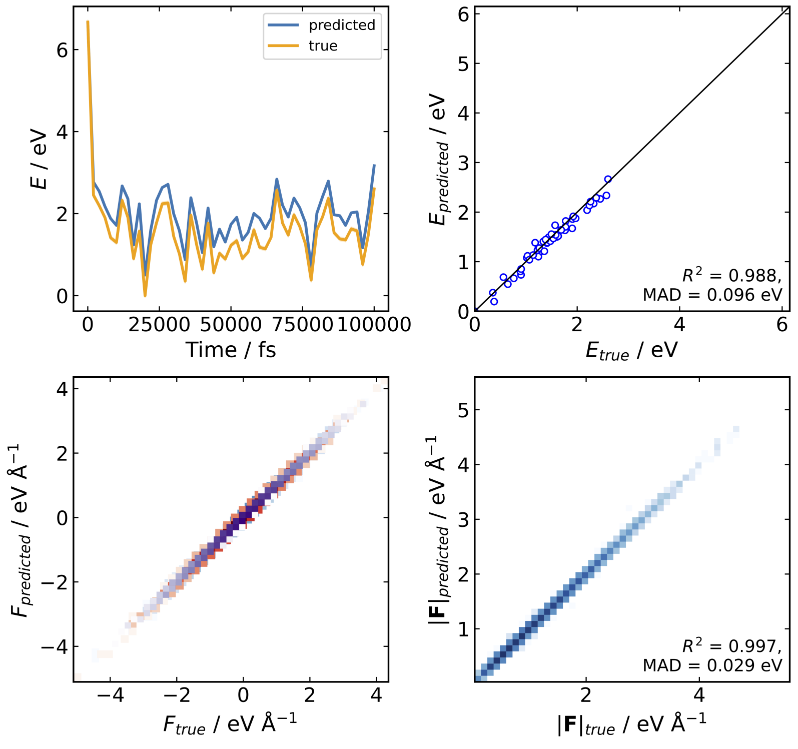

Figure S3: Comparison of ground-truth (ωB97X-D3BJ/def2-TZVP) and predicted (ACE MLP, trained after third phase of AL) energies and forces over 100 ps trajectory of the cluster containing Mg^{2+} in 46 H_2O molecules.

S2.4 Prediction error on structures with dissociated H_2O from the first solvation shell

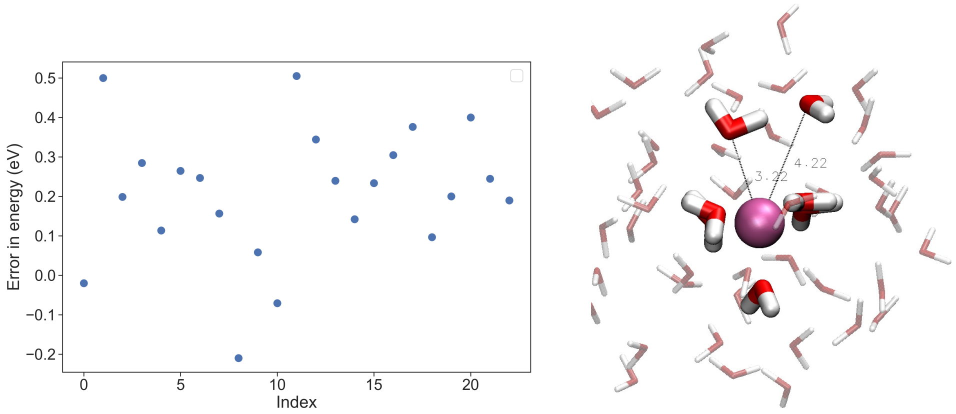

Figure S4: Energy error prediction error for 23 structures isolated from NVT dynamics after the third phase of AL.

S2.5 Artefacts from the ACE potentials for the Pd^{2+} system

A preliminary ACE potential trained on 147 data points obtained from subsequent AL steps (see section § 2.3.2 in the main text) has been tested by running molecular dynamics simulations with periodic boundary conditions (PBC). The simulation was run for 100 ps in the NVT ensemble. The starting structure for the simulation is the same one used in the next stages of this work and it has been obtained with the procedure described in the subsection § 5.2 of Computational Details in the main text. The trajectory has shown large voids around the Pd^{2+} metal ion at the axial positions of the complex. The absence of axial interactions in the complex can be observed from the Pd-N radial distribution function (Fig. S5), where the shoulder with the beginning at 2.5 Å and peak around 3.3 Å, typical of the Pd-N axial interactions, is absent.

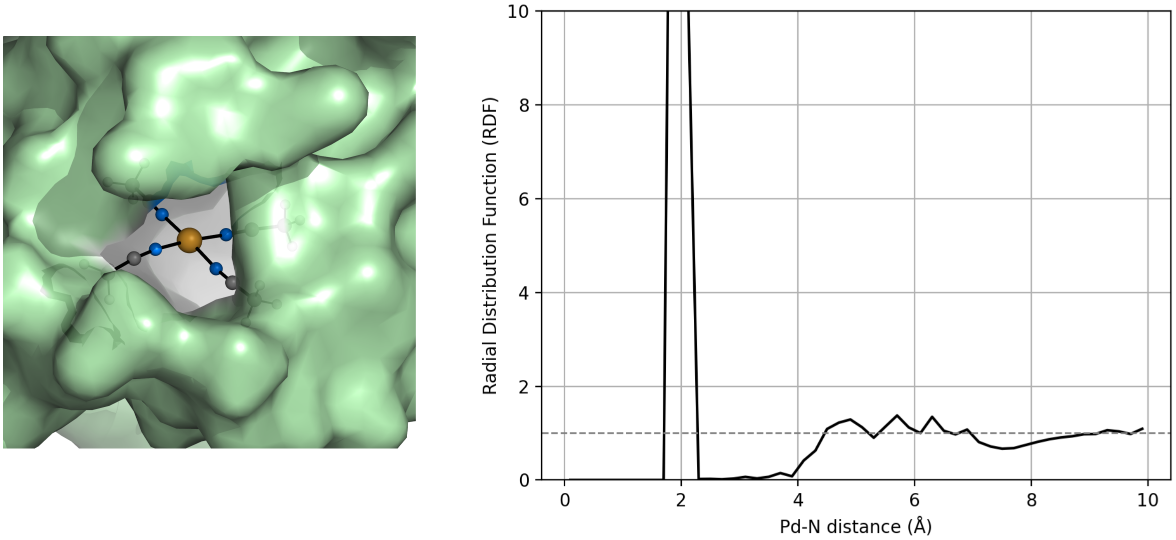

Figure S5: On the left, a representative snapshot from the 100 ps NVT with ACE showing the formation of voids around Pd^{2+}. The MeCN solvent is represented as the solvent-accessible surface. On the right, the radial distribution function from the same trajectory, evidencing the persistence of the voids around Pd^{2+} throughout the trajectory.

S2.6 Transferability of the MACE potential for Pd^{2+} in MeCN to simulating pure MeCN

The final data set for the training of the MACE potential for Pd^{2+} in this work does not contain the data of pure MeCN. For this reason, we tested the transferability of the potential towards a pure MeCN system. An NPT-equilibrated (ρ=776 kg/m^3) cubic box (edge length equal to 26.672 Å) of acetonitrile was obtained using the force field parameters from Caleman *et al.*[1]. The NVT simulation with the MACE potential was run for 250 ps. The N-N radial distribution function is reported in Fig. S6 and compared to the experimental one.[2]

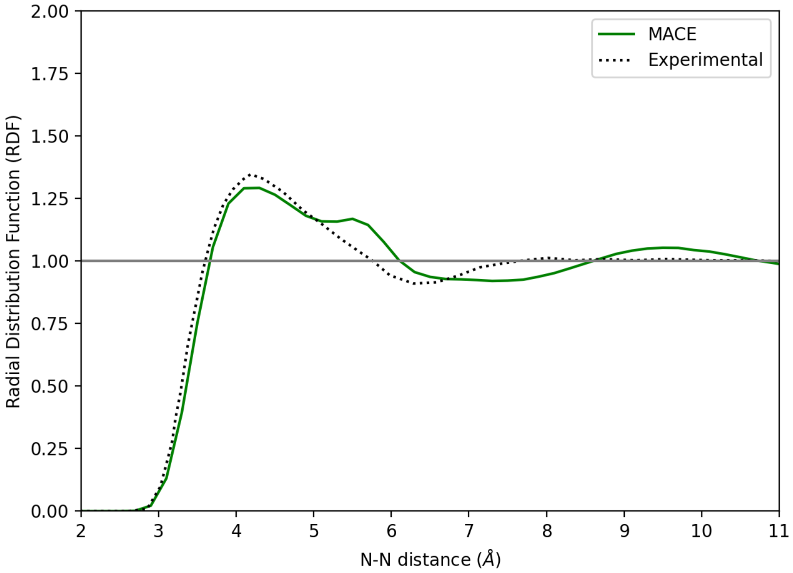

Figure S6: N-N RDF of liquid acetonitrile. Comparison between the reference experimental curve and the RDF obtained with the final version of the MACE potential from 250 ps simulation in NVT

S2.7 Stability in the NVE dynamics

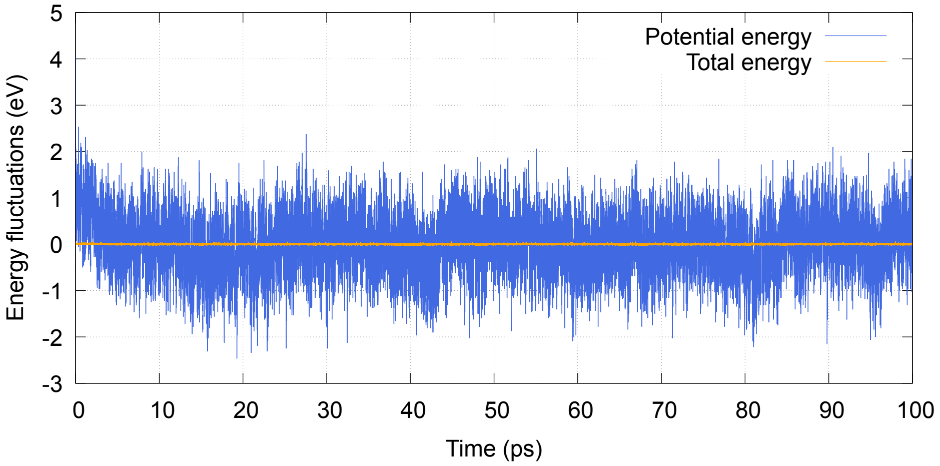

Figure S7: Potential and total energy fluctuations along 100 ps NVE dynamics of Mg^{2+} in 16.3 Å water box.

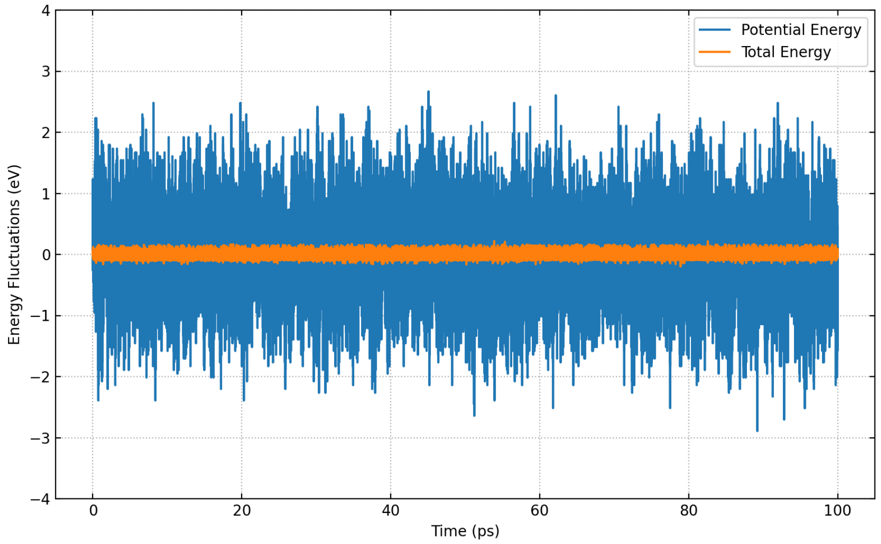

Figure S8: Potential and total energy fluctuations along 100 ps NVE dynamics of Pd^{2+} in 24.0 Å MeCN box.

The distribution of the total energy fluctuations in Fig. S8 is centred at $6.1 \cdot 10^{-9}$ eV with a standard deviation of 0.051 eV, confirming the stability of the MACE potential.

S3 RDFs and structural information

S3.1 Pd^{2+} in MeCN

The axial ligands have been identified as the ones with the closest and the second closest Pd-N distances (apart from the four equatorial ligands) that have an angle smaller than 130° with each of the four Pd-N coordinative bonds in the square planar motif (i.e., the first solvation shell). In other words, we are defining a cone with its apex on Pd and a half-angle of 40°. This selection criterion has been inspired by a previous work that investigated Pd^{2+} in liquid ammonia[3]. The plots in Fig. S9 and Fig. S10 indicate the asymmetry in the average Pd-N distances of the two axial ligands. In order to have further insights and more data about the coordination motif of the Pd^{2+} in MeCN, two independent NVT simulations of 250 ps have been performed. The two trajectories have been merged to the previously obtained NVT trajectory of 500 ps, yielding a total sampling of 1 ns. From these data, the aim was to elucidate if the average coordination motif of Pd^{2+} in MeCN is more of a distorted octahedron or a square pyramid. In order to differentiate between these two geometries, a criterion based on Pd-N distance and van der Waals radii was employed[4]. The distorted octahedron includes structures where both the closest and second closest MeCN ligands have a Pd-N distance lower than 3.75Å, while the square pyramid coordination motif includes the structures where the closest axial ligand has a Pd-N distance lower than 3.75 Å and the second closest having a longer Pd-N distance. With this criterion, in 53% of the frames, the coordination motif of Pd^{2+} is a square pyramid and in 31% of the frames it is octahedral.

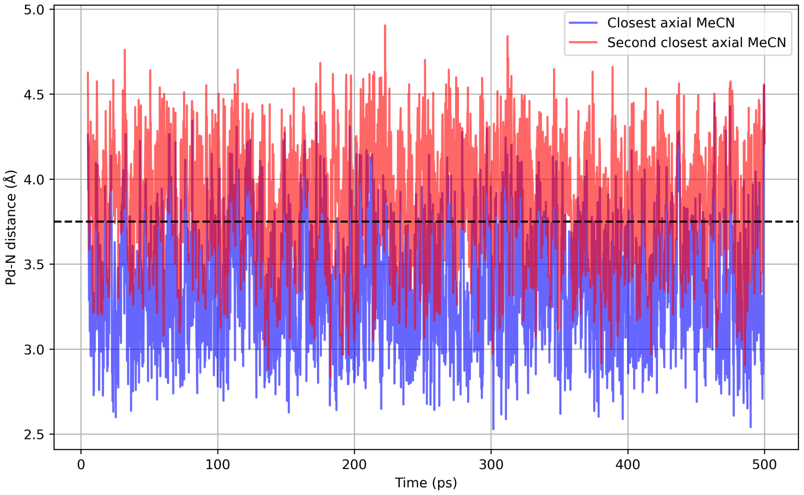

Figure S9: Time evolution of the Pd-N distances of the two axial ligands

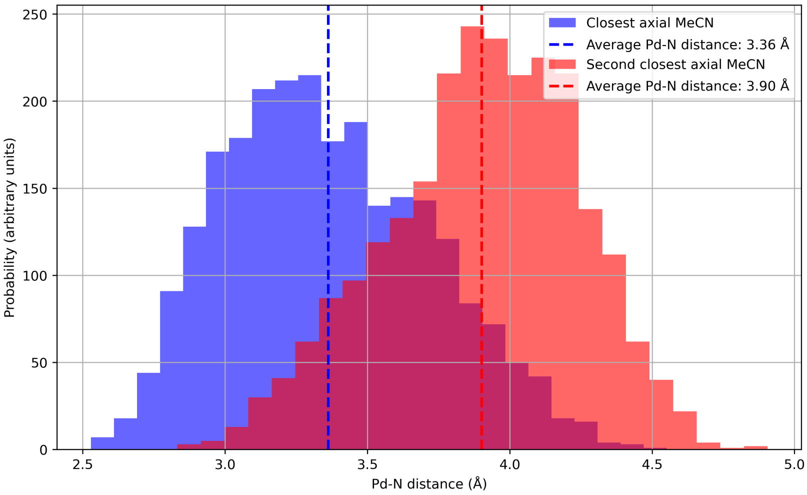

Figure S10: Distributions of the Pd-N distances of the two axial ligands

S4 Umbrella Sampling simulations

S4.1 Mg^{2+} system

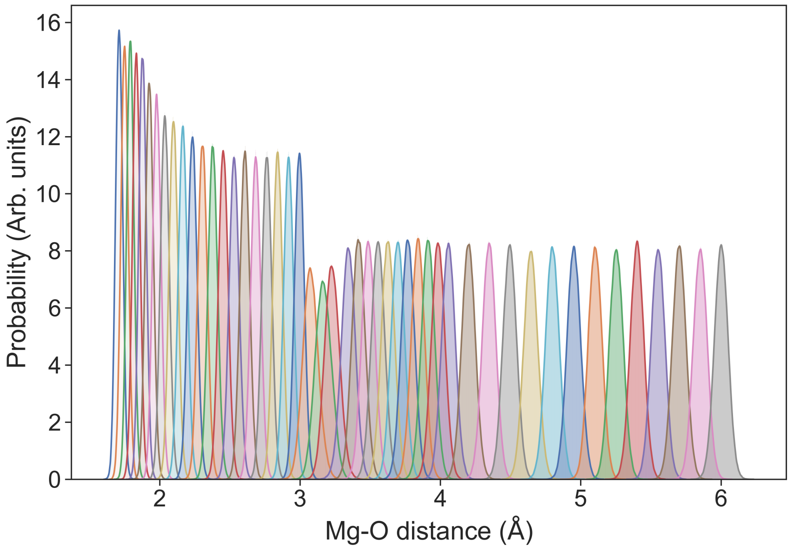

Figure S11: Histograms of the umbrella windows along Mg-O distance reaction coordinate for the first PMF.

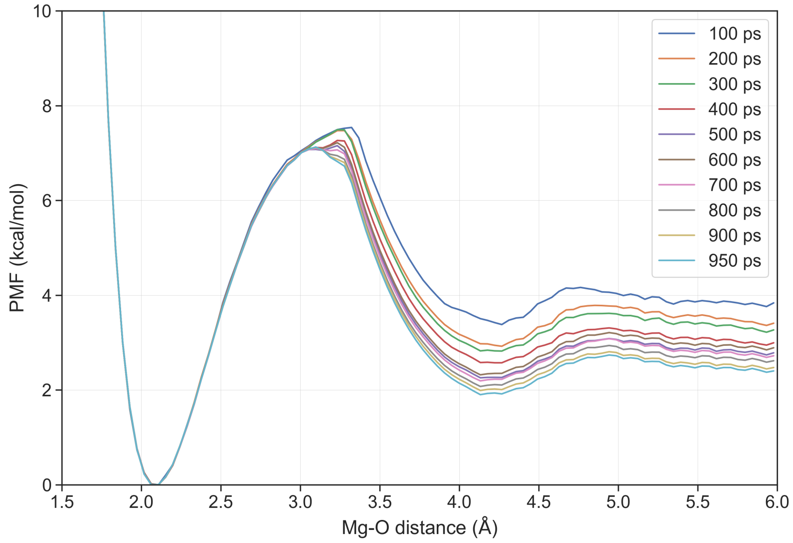

Figure S12: Convergence of the first potential of mean force as a function of the length of sampling in one window.

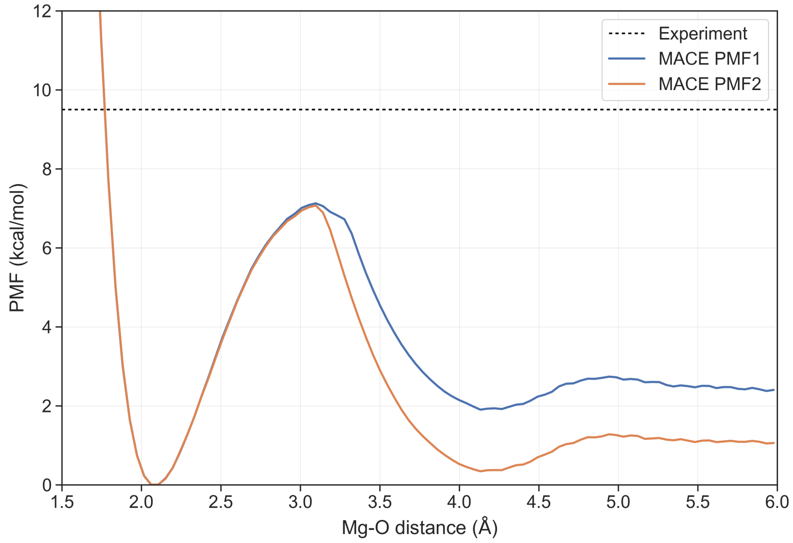

Figure S13: Convergence of the potential of mean force from two independent runs.

S4.2 Pd^{2+} system

The US simulations have been performed employing the coordination number (CN) as a collective variable (CV). The function describing the CN is

$$\mathrm{CN}(x) = \frac{1 - (\frac{x}{r_0})^n}{1 - (\frac{x}{r_0})^m} \tag{1}$$

with r_0 = 2.35 Å, n=40 and m=50. The function is defined for each of the four Pd-N bonds of the square planar complex and additionally for the Pd-N bond to a fifth MeCN in the axial position. This choice of parameters ensures that for CN=4 we have a square planar complex with the fifth axial ligand being free to move to the second solvation shell (and further towards bulk solvent), while for CN=5 the axial ligand is part of the square planar complex evolving towards the pentacoordinated transition state with a trigonal bipyramidal geometry. The function is shown in Fig. S14. The starting structures for the US were generated by steered MD: from an equilibrated frame, one of the two MeCN axial ligands was pushed towards the Pd center, guiding the system towards the formation of the pentacoordinated transition state (TS). The CN was varied between 3.98 and 5.000 in the US (Fig. S15). The spacing between the US windows varies along the CV, the centres of the windows are the following: CN = 5.000, 4.950, 4.900, 4.850, 4.800, 4.750, 4.700, 4.650, 4.600, 4.550, 4.500, 4.450, 4.400, 4.350, 4.300, 4.260, 4.220, 4.180, 4.140, 4.100, 4.070, 4.040, 4.020, 4.000, 3.980. For each window, the simulation was run with a harmonic umbrella restraint with force constant 2400 kcal/mol for 57.5 ps, using the last 50 ps in the analysis. The convergence of the free energy surface (FES) was tested by considering progressively longer portions of the trajectory for each window, see Fig. S15.
The final PMF was reconstructed using the Weighted Histogram Analysis Method (WHAM) code version 2.0.11.[5] in the range between CN=3.985 and CN=4.970 (with 0.005 spacing). The lower boundary was chosen in order to show that for CN=4 we have a minimum: further reducing the coordination number results in a steep increase in free energy since one of the four Pd-N bonds is being elongated. The choice of the upper boundary is more delicate. The CN is a convoluted collective variable that condenses the whole coordination geometry of the Pd^{2+} complex in one number. To decide which is the precise CN that corresponds to the TS, we analysed the fluctuations of the CN and the Pd-N bond lengths in the trajectory in the US window centred at CN=5.000 (Fig. S16). The CN fluctuates around a mean value of 4.96 with a standard deviation σ_{CN} of 0.01. For this reason, the TS is considered to be located at CN=4.96, yielding a barrier of 15.7 kcal/mol.

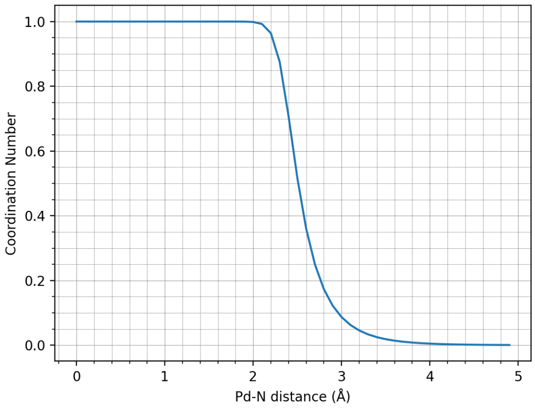

Figure S14: Coordination Number function for the Pd-N bond

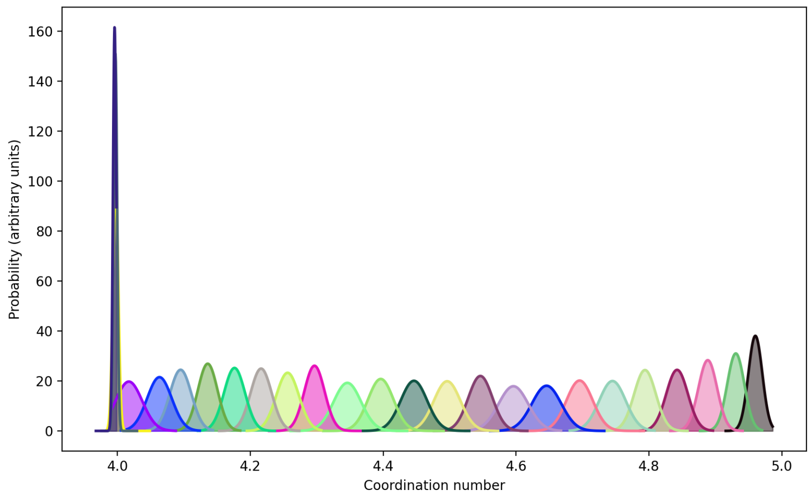

Figure S15: Histograms windows from one of the three independent US simulations for the Pd^{2+} in MeCN system.

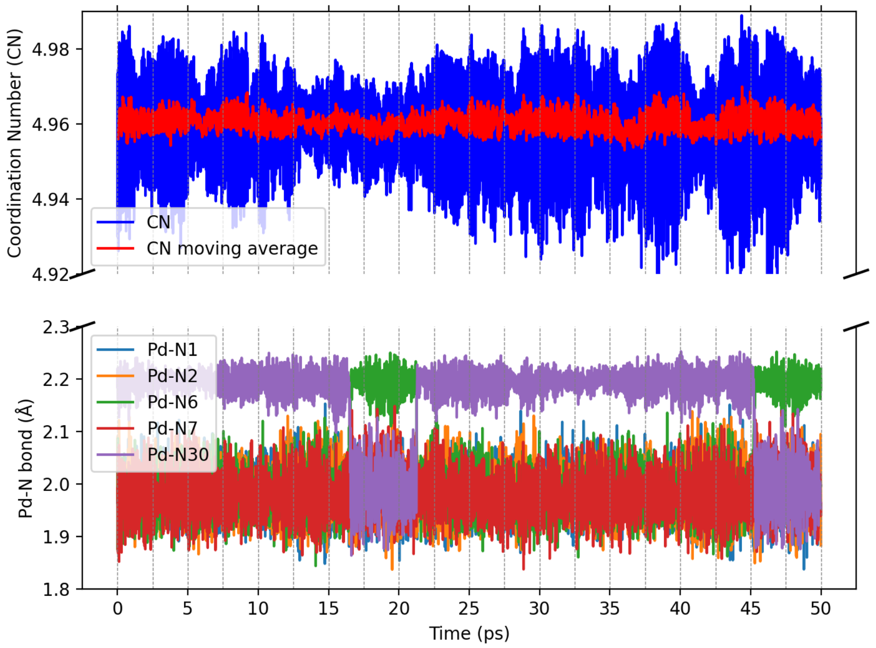

Figure S16: Trajectory from US at CN=5.000. The fluctuation and the moving average of the CN (moving average window of 100 data points, i.e. 50 fs) are plotted in the upper part of the plot. The lower plot shows the fluctuations of the five Pd-N bonds of the complex, including the ligand exchange events.

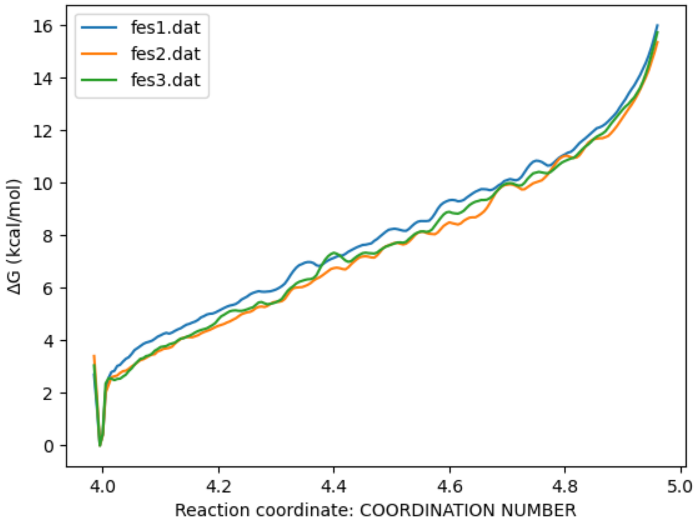

Figure S17: PMF convergence (from one of the three independent US runs) with increasing sampling time per window used in the computation of the PMF.

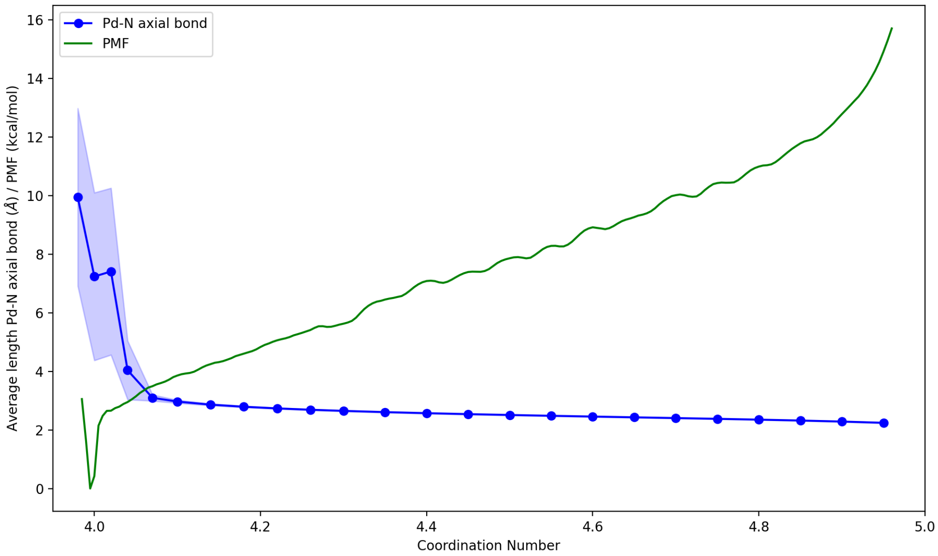

Figure S18: The Pd-N distance of the fifth MeCN ligand that participates in the ligand exchange is plotted along the PMF (averaged from the three independent US simulations). The dots represent the average Pd-N distance and the shaded area the standard deviation.

References

[1] C. Caleman, P. J. van Maaren, M. Hong, J. S. Hub, L. T. Costa and D. van der Spoel, *J. Chem. Theory Comput.*, 2012, **8**, 61–74.

[2] E. K. Humphreys, P. K. Allan, R. J. Welbourn, T. G. Youngs, A. K. Soper, C. P. Grey and S. M. Clarke, *The Journal of Physical Chemistry B*, 2015, **119**, 15320–15333.

[3] M. Saleh and T. S. Hofer, *Dalton Trans.*, 2017, **46**, 9630–9638.

[4] S. S. Batsanov, *Inorganic materials*, 2001, **37**, 871–885.

[5] A. Grossfield, *WHAM: the weighted histogram analysis method*, http://membrane.urmc.rochester.edu/wordpress/content/wham.
